# Combat and Operational Stress Control: Application in a Burn Center

**DOI:** 10.3390/ebj5010002

**Published:** 2023-12-29

**Authors:** Jill M. Cancio, Leopoldo C. Cancio

**Affiliations:** US Army Institute of Surgical Research Burn Center, 3698 Chambers Pass, Houston, TX 78234, USA; leopoldo.c.cancio.civ@health.mil

**Keywords:** combat and operational stress control, occupation-based intervention, occupational therapy

## Abstract

Occupational therapy has been integral to the holistic recovery of soldiers since its origin. The positive psychosocial and physiological effects of occupation-based interventions, fundamental to the profession, have long justified its relevance to the military. As such, occupational therapy has been written into US Army doctrine as an integral component of the Combat and Operational Stress Control (COSC) program. The focus of a COSC unit is to prevent, identify, reduce, and manage combat and operational stress reactions resulting from physical and mental stressors in a combat environment. COSC centers around the recognition and resolution of functional problems and the development of enhanced coping skills. Recognizing that burn patients are, like combatants, also at high risk of stress-related illness, we applied COSC concepts to peacetime burn care. In this paper we describe the theoretical basis for COSC in a burn center. The COSC model supports holistic, functional recovery of the burn casualty and can augment psychosocial recovery, particularly in times of limited resources.

## 1. Combat and Operational Stress Control: Application in a Burn Center

Burns are sudden, potentially life-changing injuries that may have profound and lasting effects on an individual and their family’s lives. Due to substantial improvements in burn care over the past several decades, survival after severe burn injury has dramatically increased. However, mortality [1] only captures a small aspect of the burn experience. Active engagement in life through participation in meaningful occupations and activities to achieve wellness and a sense of purpose is an often-overlooked component of post-burn recovery. Burn rehabilitation plays a significant role in the overall recovery of the burn survivor, and occupational therapy provides a specific focus on return to function.

Historically, burn rehabilitation has been closely linked to the biomechanical frame of reference. The prevention of burn-scar contracture, management of edema, and initiation of early mobility are often recognized as the primary focus areas of burn rehabilitation [2]. Less thoroughly appreciated are the psychosocial and wellness aspects of recovery. The World Health Organization [3] recognizes that burn injuries often result in both lifelong physical and psychological scarring [4,5]. The deterioration of function following aggressive physical rehabilitation that lacks psychosocial intervention means that the burn survivor’s functional recovery is significantly influenced by their mental health [6,7,8]. This suggests the importance of psychosocial recovery along with physical recovery.

Indeed, recent literature from the burn-care community asserts a need for improving psychological outcomes for burn survivors [9]. Despite this call, the delivery of psychosocial support may be difficult because of staffing issues or other priorities such as wound care [10,11]. To help meet this need, we proposed that holistic interventions, derived from occupational therapy and incorporated in the US Army’s Combat and Operational Stress Control (COSC) model, could enhance and augment the psychosocial care of burn survivors. The purpose of this paper is to describe the theoretical basis for how occupational therapy and COSC concepts can be utilized to enhance the psychosocial recovery of burn survivors. 

## 2. Combat and Burn Environments

Combat service is often associated with adverse psychological consequences. Evidence shows an increase in the risk of suicidal ideation and post-traumatic stress disorder (PTSD) among military personnel that deploy to a combat zone [12]. Of the most prominent psychological consequences, PTSD appears to be fundamental, and is a predictor of depression, anxiety, and comorbid disorders [13]. Similarly, PTSD is common in the burn population [14]. The diagnosis of PTSD requires an exposure to a traumatic event, a common feature of both combat and burn injury [12,15].

This problem is rooted in the stressors of the combat or burn environments. “In a combat situation stress can be attributed to a plethora of physical, environmental or psychosocial concerns from an enemy’s deliberate actions designed to kill, the natural environments extreme temperatures, or a leader’s decisions… Some of the most potent stressors are interpersonal in nature and can be due to conflict in the unit or on the home front” [16]. Burn casualties undergo the stress of one of the most painful injuries experienced by humans. Associated with the stressors of a burn injury, there are distinctive pre-existing psychosocial and socio-economic stressors characteristic of the burn population. Approximately one-third present with a pre-existing psychiatric diagnosis, is unemployed, has an active history of substance abuse, or has experienced a significant life event six months to one year prior to their burn injury [17,18]. Therefore, there is a well-defined need for behavioral health support in this setting.

## 3. Operational Definitions

Definitions of occupation, occupational therapy, and COSC will be provided in the following section.

### 3.1. Occupation

Occupations are meaningful and purposeful activities that individuals do to occupy their time and attention [19]. Occupation can be understood as the active or “doing” process of a person engaged in goal-directed, intrinsically gratifying, and culturally appropriate activity [20]. As a *means*, occupation brings about a transformation by the client’s participation in activities and, as an *end*, occupational participation results in an outcome of the therapeutic process [21]. Further deconstruction of occupation identifies the concepts of purpose, an organizing feature of occupation; and of meaning, the motivational and always purposeful aspect of an occupation [21,22]. 

### 3.2. Occupational Therapy

Occupational therapy is based on the premise that occupation is a health-restoring measure [23,24,25]. A philosophical underpinning to occupational therapy is the belief that a departure from daily life roles and routines attributed to reduced functional performance can lead to distress, diminished sense of purpose, and weakened self-identity [26]. Occupational therapists (OTs) assess for a functional diagnosis to design interventions that address functional deficits [27]. Occupational performance, by providing a sense of purpose and meaning, is a means to mental and physical health [16,28].

### 3.3. Combat and Operational Stress Control

Active-duty occupational therapists in the US Army receive training specific to COSC and have a specific deployment mission as members of the COSC team. The goal of COSC is to “return soldiers to duty expeditiously” [16]. The demands of the combat environment can impact functional performance; this is the impetus for providing expedient behavioral-health services on the battlefield and led to the formation of the COSC detachment [29]. The COSC mission aims at prevention, identification, and management of combat and operational stress reactions (COSRs) as they emerge. COSRs are the physiological, psychological, intellectual, and behavioral responses of service members exposed to stressful combat events or other military operations [30]. Other descriptive terms used in the past to describe COSR include battle exhaustion or fatigue and shell shock. Members of the COSC team may include a psychiatrist, social work officer, clinical psychologist, psychiatric nurse, occupational therapist, and enlisted specialists. For the purposes of this paper, we will focus on the occupational therapist’s role on the COSC team and how those skills can be applied in a burn center as a complimentary addition to the behavioral health mission. Occupational therapists are exposed to behavioral health populations in practice, and their school curriculum includes behavioral-health-specific training to include treatment techniques and options for this patient population. In addition, Army OTs are required to complete the COSC and the Management of Combat Stress Casualties courses before deploying.

## 4. Development of the COSC Model

The US military has provided a version of what is now termed combat stress control since the Revolutionary War. Countries such as Russia, France, and England have also practiced behavioral health interventions far forward on the battlefield [31]. In 1989, the US Army renamed its battlefield behavioral health units and detachments “Combat Stress Control” (CSC) units [31], and, since then, has deployed such units to prevent, reduce, identify and manage COSRs [31,32]. In 1990, MAJ Mary Laedtke was the first occupational therapist assigned to a Combat Stress Control Team and was deployed to Operation Desert Storm [30]. Occupational therapists have demonstrated and described the positive effects of occupation-based interventions, the foundational premise of the profession, and, in so doing, justified the relevance of occupational therapy as a military asset [26,29,30,32,33,34,35].

COSC interventions are defined as ‘functional areas’ and exist within either the prevention team or the fitness team [16]. The functional areas include unit needs assessment; transition management; consultation and education; traumatic-event management; restoration; triage and stabilization; reconstitution support; reconditioning; and behavioral-health treatment [16]. Viewed as a whole, COSC interventions [16] transition through all three stages of the preventive health continuum to include primary, secondary, and tertiary prevention. These levels of prevention are described by Reitz et al. [36]. A comparison of interventions provided at a COSC unit and a burn center is listed in Table 1.

The performance of functional-area tasks requires identifying risks and/or COSRs through individual soldier or unit information and objective COSC findings, and preventing or managing those problems. Therapeutic interventions focus on the recognition and resolution of problems, enhanced coping skills, and integration of skills into action to optimize human performance when returned to the battlefield [16,26,29]. 

To achieve successful outcomes, personnel within the COSC apply six management principles: brevity, immediacy, contact, expectancy, proximity, and simplicity (BICEPS) [16,37]. These management principles are described in Table 2. The management principles are applied in each functional area to enhance adaptive stress reactions, prevent maladaptive stress reactions, control COSRs and behavioral-health disorders, and teach resiliency skills [16]. The COSC program optimizes performance; protects the will to survive; and averts adverse physical, psychological, intellectual, and social health effects [16,26,37]. To facilitate these outcomes, COSC employs the targeted use of activity to maximize life skills [16,26,37,38]. 

Identified experientially from various combat environments are the costs of uncontrolled combat and operational stress: “erratic or harmful behaviors… impair[ed] mission performance... [and] failure” [16]. The goal of COSC interventions are to optimize performance; protect the will to survive; and prevent adverse physical, psychological, intellectual, and social health effects [16,26].

## 5. Effectiveness of COSC in Operational Environments

There are few studies that evaluate the effectiveness of mental health treatments in a deployed setting. The primary aim of a COSC unit is to help prevent behavioral health problems, preserve combat power, and increase return to duty rates for combat-stress-related casualties as close as safely possible to a hostile environment. A study conducted in Iraq evaluating the clinical effectiveness of a brief treatment program designed to address combat stress reactions (CSRs) in soldiers suffering from deployment-related stressors was reported to be the first study to do so in a deployed environment [39]. Although there were associated limitations with regard to study design to include challenges of conducting research in a deployed setting, this study provided preliminary data to support the utility of the program and its benefits for service members in a deployed setting as evidenced by a statistically significant decrease in post-traumatic stress disorder (PTSD) symptoms after the completion of the program [39]. 

Another paper published in 2017 reported on a process improvement project to determine the effectiveness of a COSC Center in Afghanistan. Service members were referred to a Freedom Restoration Clinic (FRC) for participation in a three-day program [40]. The officer in charge of the FRC was an Army OT and clinical support was provided by various members of the COSC clinic consisting of a psychiatrist, clinical psychologist, and clinical social worker. The OT personnel lead the groups (top five groups included anger management, resiliency, goal setting, stress management, and positive thinking), activities, physical training, and individual sessions as needed. Short-term follow-up after completion of the program yielded significant improvement in stress-related symptoms compared to prior to initiating the program, but, after 30 days, the results were no longer significantly improved.

It is important to mention that these studies lack long-term follow-up data regarding program effectiveness. Consideration of additional measures of acute stress disorder, anxiety, or depression to assess other aspects of symptom change may also be beneficial. A systematic review and meta-analysis published in 2021 indicated that, although COSC interventions may play a valuable role in decreasing stress, decreasing absenteeism, and enabling return to duty, there is little evidence that suggests an overall effectiveness in the prevention of PTSD in military service members [41].

## 6. Integration of COSC into Burn Rehabilitation

The COSC model has demonstrated success in behavioral-health symptom reduction in active-duty service members, in large part through the use of holistic occupational therapy interventions [39,40]. Considering the success of this program and the similarities between combat and burn survivors with regard to PSTD, depression, and anxiety, our Burn Center leveraged COSC-trained occupational therapists to enhance its behavioral-health services. This was of particular importance at a time when our Burn Center was without a psychologist due to staff turnover. The goal was not to replace but rather to augment psychological and psychiatric care. The application of the COSC model was guided by the idea that optimal recovery from injury requires the employment of a person’s need for engagement in functional tasks. Moreover, as referenced in Table 2, we use the term “support network” which expands beyond friends and family and represents other important support people such as clergy members, employers, and support groups.

This approach aims to address both the acute and chronic aspects of a burn injury. A major burn injury meets the definition of a chronic disease, as there is potential for a decline in mental and physical health over time [4,5,42]. Therefore, occupational therapy addresses the chronic effects of a life-changing injury, incorporating holistic concepts that promote self-care, mental resilience, and lifelong adaptive skills. The primary goal is to restore health, and to instill wellness and the need to ‘do’ and to ‘be,’ as a means to adapt and belong [21,24,25,43]. The application of the COSC approach can extend beyond care in the acute hospital setting. For example, as referenced in the previous section, as part of the COSC program in Afghanistan, occupational therapists provided group treatment sessions to include topics such as anger management, resiliency, goal setting, stress management, and positive thinking. Other group topics might include spirituality and community integration. These groups can be made available to patients who are still in the hospital, as well as adapted to those who have progressed to outpatient rehabilitation or come to the burn clinic. Logistics regarding infection control may need to be considered; however, viewpoints from patients in the acute stages of recovery verses more sub-acute or long-term stages of recovery may provide varying experiences that benefit other members of the group.

### 6.1. COSC Focus on Recovery

The COSC model focuses on four stages of functional recovery: (1) the recognition of functional problems; (2) the development of enhanced coping skills; (3) performance using those skills; and (4) the resolution of problems to enable an expedient return to duty. Whether in a COSC detachment or the Burn Center, the occupational therapist implements an assessment that specifies the problem and a functional diagnosis, directs the construction of effective adaptive skills, and applies the interventions through occupational engagement. Performance that achieves problem resolution and a successful outcome is realized as the optimization of human performance. In so doing, the cardinal rules for OT intervention as described by Dunton [44] are that: (1) interventions must be curative; (2) they must be interesting and useful, and encouragement must be given; (3) they must increase knowledge, must be performed in groups, and must not end in fatigue; and finally, (4) a poor product is better than idleness. 

### 6.2. Applying the Six COSC Management Principles in a Burn Center

Next, we will describe the six management principles of COSC (Table 2) as applied in burn center care by occupational therapists. 

*Brevity* within COSC means efficiency in time utilization for the recovery of physiological resources [16]. The expectation at COSC facilities is that rest and replenishment is located close to the soldier’s unit and should last no more than one to three days [45]. This approach uses the “5 R’s.” The casualty is (1) reassured that their response is normal; (2) rested from combat or operational demands; (3) replenished of physiological needs such as water, food, hygiene, and sleep; (4) restored with confidence in the successful performance of activities; and (5) returned to duty [16,37,45]. 

The concept of brevity is associated with William Dunton’s [44] rule that interventions “must not end in fatigue,” as fatigue risks deteriorated quality of performance [31,35]. Interventions implemented to improve the opportunity for brevity and coincide with the five R’s within the Burn Center are: (1) assurance that the individual response to a harmful experience is normal; (2) pain management to decrease emotional and physiological demands from the trauma of wound care and functional recovery; (3) sleep hygiene, adequate nutrition, and wound healing; (4) when appropriate, the spacing and timing of painful procedures such as wound care, surgical procedures, and intense range of motion (ROM) sessions to mitigate re-traumatization and microtraumas (i.e., wound care every other day if appropriate or simultaneously pairing painful procedures such as wound care or surgery with ROM treatment sessions when analgesia coverage is greater to decrease the amount and duration of painful procedures experienced during the day); (5) the simulation of premorbid daily routines to include maximizing activity-of-daily-living (ADL) performance; and (6) discharge planning initiated on the day of admission and continued daily, with an understanding that the injured person is expected to recover and leave the hospital. 

*Immediacy* is the rapid implementation of interventions. This is a well-established and executed principle in COSC and the Burn Center. In both environments, the casualty is evaluated by rehabilitation services within 24 h of arrival; intervention is initiated immediately thereafter. This reduces the risk of further dysfunction and instills the concept of role retention (e.g., COSC casualties are identified as soldiers, Burn Center casualties are identified by their premorbid functional capabilities). Immediately following a traumatic event, a person may not want to engage in functional recovery. At that time, an activity intended to promote functional recovery may not look ideal, but it is effective as a reference point as progress is made. 

*Contact* refers to the socialization and social identity of the individual within their group. A COSC casualty remains engaged with unit leadership [16]. The Burn Center casualty has communication restored with family, friends, and other identified members of their support network (i.e., work colleagues, or religious or spiritual support people) as quickly as possible. Conceptually, the individual retains their pre-injury identity, avoiding the patient identity and role. Contact supports an emotionally healthy perspective, and its significance has been identified as instrumental in overall functional outcomes. Emotional well-being from the point of injury has been identified as more significant to the functional outcome of a burn casualty than the physiological trauma [8,46]. 

*Expectancy* continues the concept of a positive attitude through working with the burn survivor. Daily care is related to an understanding that the injured person is expected to recover and be discharged from the hospital with the ability to experience daily routines [16,38].

*Proximity* associates the intervention physically close to the individual’s military unit for COSC recovery [16]. For the burn casualty, the need for care requires hospitalization at the specialized burn center for an undetermined amount of time. Proximity is achieved through connection with family, friends, and coworkers using phone calls, video calls, or physical visits from those who can travel to the Burn Center. Often, these opportunities require problem solving and planning. The burn casualty will be required to engage in the process.

*Simplicity* is critical to both environments of care and is foundational to the curative effect one can have with therapeutic interventions. The closer an intervention is to what one typically does, the closer one is to being more compliant. This is where occupation and the concepts of meaning and purpose are applied. If the activity is meaningful, it fulfills a purpose for the person performing the activity. Occupation, in this sense, is a change agent, creating the opportunity for goal achievement, competency, and self-esteem [21]. In the Burn Center, occupational therapists can apply the concept of occupation-based care which allows the burn survivor to engage in real-life situations that match their interests and needs, thereby developing a sense of belonging and return to normalcy. 

### 6.3. COSC Functional Areas

COSC interventions are organized into nine functional areas [16,45]. The purpose of these functions is to cover all components of behavioral health care from preventative through clinical intervention. This allows the COSC team to identify the specific areas of COSC need, discover contributory factors impacting these needs, provide an assessment of the behavioral health training needs, and develop plans to meet or improve the COSC needs of the soldiers and units. Table 3 provides a summary of the nine functional areas and their application to the burn center. The five R’s of recovery listed previously are also incorporated into the application of COSC functional areas into burn-center operations.

## 7. Program Objectives

The following section provides a description of program objectives employed by occupational therapists at the US Army Burn Center as adapted from the COSC mission. 

### 7.1. Enhance Adaptive Stress Reactions

This is the process to promote enhanced adaptive stress reactions using occupational engagement. The activity is constructed as a means to achieve the goal. Occupational performance is used to enhance adaptive stress reactions to reduce the vulnerabilities to disorders such as anxiety, depression, and addiction that may develop [47]. This concept coincides with operational practice in COSC [16,26]. At the US Army Burn Center, enhanced adaptive stress reactions are influenced through occupational performance on a continuum of modifications to include graded and/or adaptive techniques to achieve success in activity performance.

### 7.2. Prevent Maladaptive Stress Reactions

As in the COSC environment of care, the US Army Burn Center occupational therapists implement the process to prevent maladaptive stress reactions using occupational engagement. Therapeutic activity is designed to bring about psychosocial and behavioral change through performance. Occupational therapists leverage an authentic therapeutic relationship that is built upon care, trust, and respect, in order for interventions to have the greatest impact [48].

### 7.3. Control COSRs and Behavioral Health Disorders

The most common behavioral health diagnoses seen in burn survivors are acute stress disorder, anxiety, depression, and post-traumatic stress disorder. Baseline screening of patients admitted to our Burn Center takes place within the first 72 h of admission to the Burn Center. During the absence of a psychologist, the occupational therapy team conducted these baseline screenings. Currently, the occupational therapy team continues to be available to assist the psychologist with baseline screenings as needed and notifies the team of any psychosocial concerns that may require elevated levels of care or referral to psychiatry. 

When a patient screens within the low range of risk, no additional interventions are indicated or prescribed. At this time, the individual will be monitored, particularly after surgical procedures or other potentially emotional events. When screening demonstrates moderate to high levels of neuropsychiatric disease or a diminution of the positive qualities of stress, interventions based on a stress challenge response will be introduced to the patient’s treatment plan. Stress can be detrimental when perceived as threatening or health-producing when perceived as a challenge [49]. The stress challenge is introduced to support motivation related to painful activities. The progress that results supports the fundamentals of occupational therapy, that the state of mind creates the emotional experience, and the physical performance of meaningful activity translates to an adaptation to a healthier state [23,25]. As such, the occupational therapy team provides augmented occupation-based treatments that build on the concepts employed by the psychologist. Co-treatments with the psychologist and the physical rehabilitation team are also leveraged to enhance recovery strategies, as the treatment plans and skill sets of the team members often complement one another.

### 7.4. Teach Warrior Resiliency Skills

The focus of resilience is adaptation, achieved through the application of ever-changing environmental factors, activity factors, and the assessment of occupational performance [50]. Additional practices include the application of vicarious resilience, in which one finds meaning and experiences growth (adaptation) from the traumatic experiences of others [49]. 

### 7.5. Differences between Deployed Setting and Burn Center

A soldier in a theater of operations has been vetted, trained, and indoctrinated into a unit for the purpose of a mission. There may be a shared assumption that the soldier, if injured, is likely to survive. This is not necessarily the typical mindset of the peacetime burn casualty. Furthermore, the burn survivor’s functional recovery is significantly influenced by their premorbid mental health [6,7,42]. Pre-existing psychiatric morbidity is common in burn patients. There is a close association between a psychiatric history (depression), substance abuse disorders (alcohol and drug dependence), stressful life events, unemployment, and burns [51,52,53]. These data regarding the high likelihood of concomitant premorbid mental health conditions in civilians who sustained burn injury further justify the need for and significance of COSC concepts and occupation-based care to augment the psychosocial recovery of burn survivors. It is important to articulate that there is a *complimentary* rather than competitive relationship between occupational therapy and psychology. The psychosocial recovery of the burn survivor provides a unique opportunity to leverage the skill sets of each discipline in a symbiotic manner to enhance recovery and return to function. Further quantitative and qualitative study of the holistic power of occupation in the restoration of function in burn survivors is warranted and necessary.

## 8. Conclusions

Burn rehabilitation has historically focused on a biomechanical frame of reference, addressing burn-scar contracture and the biomechanical dysfunction that follows [2,54]. Less thoroughly incorporated into burn rehabilitation is recovery from psychosocial injury. Occupational therapy has been integral to the holistic recovery of soldiers since the origin of the profession. The positive psychosocial and physiological effects of occupation-based interventions, a fundamental principle of the profession, has justified occupational therapy’s relevance as a military asset [24,30,32,33,34,35]. Occupational therapists are members of the rehabilitation team, as well as vital members of the COSC team in their deployment role. This ideally positions OTs to leverage skills from both of these contexts. There are preliminary data that demonstrate that the COSC mission has resulted in symptom and disorder amelioration in deployed soldiers at least in the short term [40,41]. It is important to understand that further study and refinement of these programs are required to assess long-term effectiveness. Nevertheless, the COSC concept does provide a viable framework to enhance the delivery of a more holistic approach to recovery that includes an emphasis on psychosocial recovery. We have proposed adapting the COSC model into burn rehabilitation. We aim to develop and employ whole-person, occupation-based interventions to achieve adaptation and to reduce the risk of functional deterioration following community reintegration [24]. Future research at our Burn Center will focus on strategies to document and improve long-term functional outcomes following burn injury.

## Figures and Tables

**Table 1:** Levels of prevention and comparison of COSC and burn center operations.

Levels of Prevention: Intervention Comparison		
Health and Wellness	COSC	Burn Center
Primary Prevention	Prevention Team: Unit needs assessment Consultation and education Transition management	Healthy Population: Decrease risk via community outreach programs focused on prevention
Secondary Prevention	Fitness Team: Traumatic event management Triage and stabilization Restoration	Acutely Injured Population: Restoration to pre-morbid condition (health) without residual effect from injury or illness Traumatic event management through surveillance screening and individual and or group treatment sessions
Tertiary Prevention	Fitness Team: Reconstruction support Behavioral health treatment Reconditioning	Chronic Disability Population: Intervention emphasizes prevention of further dysfunction from chronic condition

**Table 2:** Six management principles of COSC.

COSC Management Principles		
	COSC	Burn Center
Brevity	Rest and replenishment × 1–3 days	Modulation of sleep/wake cycle; delirium management and sleep hygiene
Immediacy	Intervention as soon as symptoms are identified	Rehabilitation evaluation within 24 h; regular monitoring of psychological symptoms
Contact	Unit contact, maintain soldier mentality (not a patient)	Regular contact with support network (physical and virtual)
Expectancy	Expected to recover and return to full duty	Return to highest level of function
Proximity	Recover in close proximity to parent unit	Involvement of support network in care
Simplicity	Brief, up-front methods	Occupation-based care to return a sense of normalcy

Adapted from ATP 4-02.8 [16].

**Table 3:** Relationship of COSC functional areas to burn center operations.

COSC Functional Area	COSC	Burn Center
Unit needs assessment	Identify needs, priorities, allocate resources	Initial evaluation, interdisciplinary communication
Restoration	Replenish psychological needs, safe environment, belonging	Sleep hygiene, ADLs, socialization
Reconditioning	Intensive work therapy, physical training, psychotherapy	Adaptation to re-establish a daily routine
Traumatic event management	Process emotional/cognitive responses of a traumatic event. Goal: enhance coping skills; return to baseline functional performance	Same
Consultation and education	Transmit information, promote interactive relationship, educate by multiple means	Communication with patient, family, friends; verbal, visual (handouts, websites, telemedicine)
Psychiatric treatment	Triage and stabilization	Intervention following stabilization based on recs from psychology/psychiatry
Reconstitution	Allows command to plan for increased combat effectiveness	Not applicable
Transition management	Gain understanding and support needs of unit and staff	Discharge planning for resources/equipment
Triage and stabilization	Sorting soldiers with COSRs and behavioral health disorders where they can best be treated	Intervention following stabilization based on recommendations from psychology/psychiatry; return to function

## Data Availability

No new data were created or analyzed in this study. Data sharing is not applicable to this article.
